# Bankline detection of GF-3 SAR images based on shearlet

**DOI:** 10.7717/peerj-cs.611

**Published:** 2021-12-22

**Authors:** Zengguo Sun, Guodong Zhao, Marcin Woźniak, Rafał Scherer, Robertas Damaševičius

**Affiliations:** 1 Key Laboratory of Modern Teaching Technology, Ministry of Education, Xi’an, Shaanxi, China; 2 School of Computer Science, Shaanxi Normal University, Xi’an, Shaanxi, China; 3 Faculty of Applied Mathematics, Silesian University of Technology, Gliwice, Poland; 4 Department of Intelligent Computer Systems, Czestochowa University of Technology, Częstochowa, Poland; 5 Department of Applied Informatics, Vytautas Magnus University, Kaunas, Lithuania

**Keywords:** Shearlet, GF-3 synthetic aperture radar images, Bankline detection, Morphological processing, Non-local means

## Abstract

The GF-3 satellite is China’s first self-developed active imaging C-band multi-polarization synthetic aperture radar (SAR) satellite with complete intellectual property rights, which is widely used in various fields. Among them, the detection and recognition of banklines of GF-3 SAR image has very important application value for map matching, ship navigation, water environment monitoring and other fields. However, due to the coherent imaging mechanism, the GF-3 SAR image has obvious speckle, which affects the interpretation of the image seriously. Based on the excellent multi-scale, directionality and the optimal sparsity of the shearlet, a bankline detection algorithm based on shearlet is proposed. Firstly, we use non-local means filter to preprocess GF-3 SAR image, so as to reduce the interference of speckle on bankline detection. Secondly, shearlet is used to detect the bankline of the image. Finally, morphological processing is used to refine the bankline and further eliminate the false bankline caused by the speckle, so as to obtain the ideal bankline detection results. Experimental results show that the proposed method can effectively overcome the interference of speckle, and can detect the bankline information of GF-3 SAR image completely and smoothly.

## Introduction

Synthetic aperture radar (SAR) is a high-resolution coherent imaging radar, as a type of active microwave imaging sensor, which can work at any time day or night and under all weather conditions. It is widely used in agriculture, disaster monitoring, geology, lakes, ocean monitoring and navigation, and other fields. The synthetic aperture radar satellites currently in orbit mainly include Canada’s Radarsat-2 (Gherboudi et al., 2011), the European Space Agency’s Sentinel-1 (Twele et al., 2016) and others. The imaging modes of these two satellites are 10 and 5 respectively, and the highest resolution is 1 and 5 m respectively. As China’s first self-developed active imaging C-band multi-polarization SAR imaging satellite with complete intellectual property rights, GF-3 has a resolution of 1 m and 12 imaging modes (Zhang, 2017). In-orbit testing in 2016 shows that the resolution, width, radiation accuracy, positioning accuracy and other indicators of the GF-3 satellite have met the development requirements, so the GF-3 satellite can provide high-quality and high-precision earth observation data for users. All the GF-3 SAR images are obtained from China Centre for Resources Satellite Data and Application, which is the national remote sensing satellite data center with the largest number of civil land observation satellites in orbit in China. The detection and recognition of banklines in GF-3 SAR image has very important application value for map matching, ship navigation, water environment monitoring and other fields. The identification of many important man-made geographic targets (such as bridges, dams, ports, etc.) are also based on bankline detection (Guo et al., 2016; Luqman et al., 2017).

Since the coherent imaging mechanism of GF-3 SAR images is easy to form speckle (Zhang et al., 2018), the traditional differential operator-based edge detection method cannot be applied to the bankline detection of GF-3 SAR images. Traditional difference-based edge detectors are represented by Prewitt operator, Sobel operator, and Log operator (Andrade-Loarca, Kutyniok & Öktem, 2020; He et al., 2020). They treat the differentiation as a representation of the edges and perform edge detection by constructing a differential operator that is sensitive to pixel gray level changes. Although this method can detect the edge information of the image, it cannot overcome the speckle effect of the GF-3 SAR image (An, Pan & You, 2018). This method often detects the false bankline mixed with the real bankline, and even submerges the coastline in SAR image, which reduces the reliability of the detection results. In Mount et al. (2013), continuous wavelet transform is used to study the morphological change of Jamuna River in Bangladesh, which can effectively extract the information of complex bankline with multi-channel crisscross. However, due to the low approximation degree of wavelet transform for features, the algorithm cannot accurately describe the characteristics of river bankline. Yun et al. (2016) proposed a bankline detection method based on principal curve extraction. The bankline detected by this method has a higher degree of smoothness and stronger continuity. However, the limitation of this algorithm is that it can only detect the information of bankline in optical image, and cannot be applied to the feature extraction of bankline in SAR image. In Li et al. (2018), different proportions of wavelet transform results are combined to achieve the purpose of accurate detection of bankline. The algorithm has strong robustness but low directional sensitivity, and cannot detect bankline in all directions of image. Table 1 summarizes the advantages and disadvantages of the three bankline detection algorithms. These three algorithms are designed for optical images, without considering the influence of speckle in SAR images, and cannot be applied to bankline detection in GF-3 SAR images. Therefore, we hope to propose a detection method which can effectively overcome the speckle interference of SAR image and accurately identify the location of the bankline. Shao, Sheng & Sun (2017) show that GF-3 SAR image has good sparsity. Therefore, only by fully considering the good sparsity characteristics of GF-3 SAR images, we can design an efficient bankline detection algorithm to obtain high-quality bankline detection results.

**Table 1:** Analysis of advantages and disadvantages of three algorithms.

Algorithm	Advantage	Disadvantage
Algorithm in Mount et al. (2013)	Extracting the complex bankline information with multiple rivers effectively	Weak ability to describe the characteristics of bankline
Algorithm in Guo et al. (2016)	Obtaining more smooth and continuous bankline	Only detecting the bankline information in optical images, and unsuitabale for SAR images
Algorithm in Li et al. (2018)	Having high accuracy of bankline extraction and strong robustness	Low direction sensitivity, and not detecting the bankline in all directions of image

In recent years, multi-scale geometric analysis (MGA) tools have been widely used in the field of image processing (De Oliveira, Carvalho-Ribeiro & Maia-Barbosa, 2020). Among them, Shearlet is the latest development of the MGA tools. It was constructed by Easley, Labate & Lim (2008) and Sun et al. (2020) through a special form of affine system with compound expansion. Shearlet is anisotropic and can capture edge information and anisotropic feature information more efficiently. Compared with other MGA tools, shearlet is more suitable for extracting geometric features from multi-dimensional data because of its optimal sparsity and stronger orientation. Therefore, shearlet has been widely used in image denoising (Easley, Labate & Lim, 2008), inverse problem (Patel, Easley & Healy, 2009), image enhancement (Patel, Easley & Healy, 2008), image separation (Fadili et al., 2010) and 3D data processing (Schug, Easley & O’Leary, 2011). In this paper, we choose shearlet to detect the bankline of GF-3 SAR image, which can better suppress the influence of speckle, improve the detection accuracy, and obtain continuous and accurate bankline detection results.

In this paper, the non-local mean filter is used to preprocess the GF-3 SAR image, so as to reduce the interference of speckle on the bankline detection. Secondly, shearlet with optimal sparsity and strong directionality is used to detect the bankline of the image. Finally, morphological processing is used to refine the bankline and further eliminate the false bankline caused by the speckle, so as to obtain the ideal bankline detection results. Compared with the existing bankline detection methods, the proposed method can effectively overcome the speckle interference in GF-3 SAR image, and the detected banklines are smooth and continuous, with high accuracy.

## Related work

### Brief introduction of GF-3 SAR image

**Figure 1:**
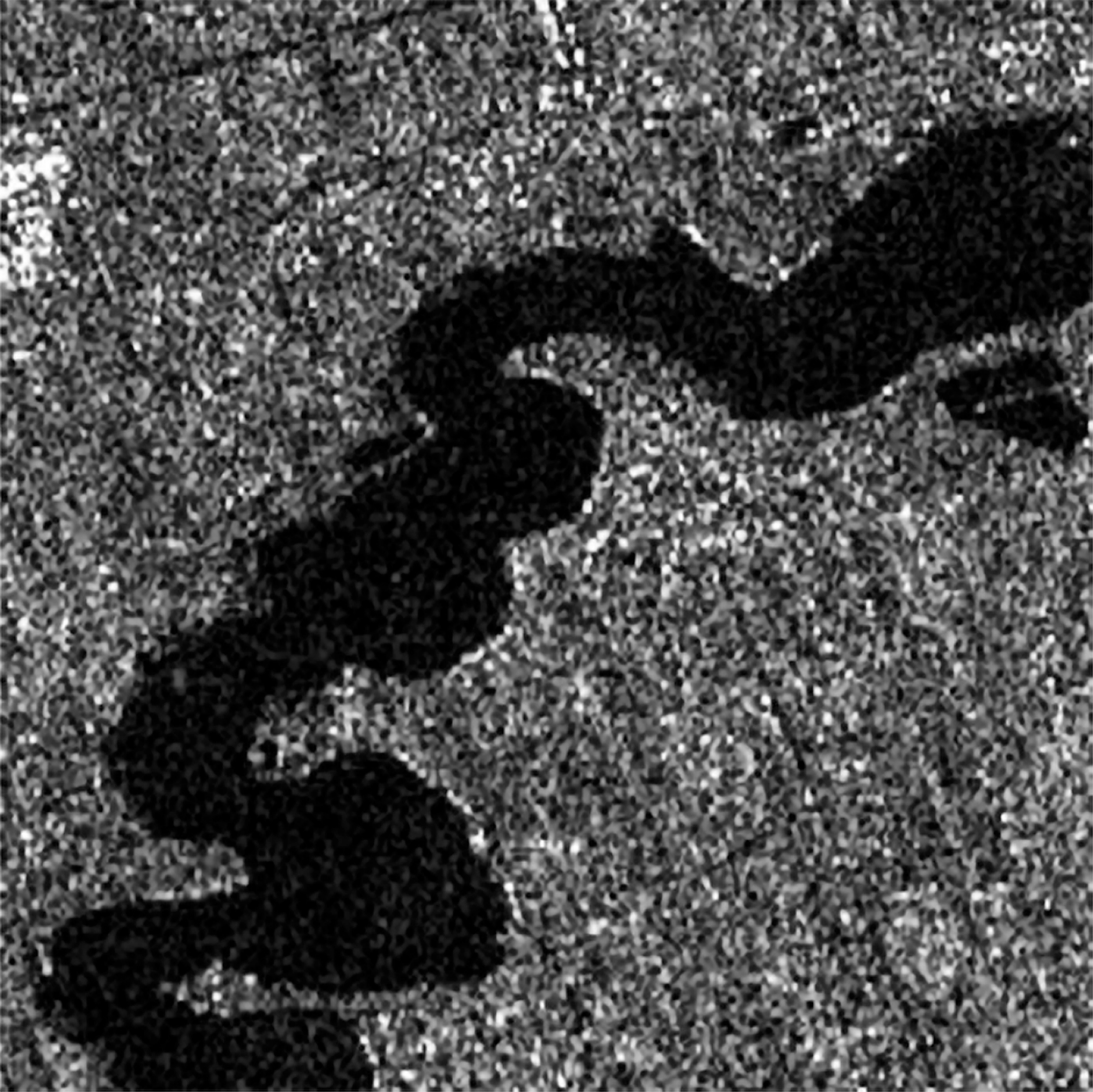
River course of GF-3 SAR image.

The GF-3 satellite is China’s first self-developed active imaging C-band multi-polarization SAR imaging satellite with complete intellectual property rights, with a resolution of 1 m and 12 imaging modes. The satellite can provide users with stable data services, greatly enhance the efficiency of the satellite system, and has a wide range of applications in various fields. Whether it is compared with the resolution of 5–20 m of Sentinel-1 satellite, or the resolution of 1–100 m of Radarsat-2 satellite, the resolution of 1–500 m of GF-3 satellite can collect more complete information of ground objects. Its image quality indexes have reached or exceeded the level of similar international SAR satellites. The index of in-orbit test shows that the absolute radiation accuracy of GF-3 (Schmidt, Ramon & Schwerdt, 2018) can reach 1.3–1.4 *dB*. The GF-3 SAR image is shown in Fig. 1, and its corresponding parameters are shown in Table 2. It can be seen from the image that the image is seriously interfered by speckle. In the image, the river is shown as a deep gray area, and the bankline is difficult to detect due to speckle. Shao, Sheng & Sun (2017) show the GF-3 SAR image has good sparsity. Therefore, we use the optimal sparsity, excellent multi-scale and directivity of shearlet to detect the bankline of GF-3 SAR image.

**Table 2:** Imaging parameters of GF-3 SAR image.

Imaging model	Polarization	Resolution	Imaging position
FS2	HHHV	10m	E108.8
			N34.6

### Shearlet introduction

Easley, Labate & Lim (2008) and Bubba et al. (2019) proposed a new multi-scale geometric analysis method based on the traditional affine system theory: shearlet. Shearlets are constructed by a special form of affine system with synthetic expansion. The two-dimensional compound expansive affine systems can be expressed as

(1) $${\Psi _{AB}}\left( \psi \right) = \left\{ {\matrix{ {{\psi _{j,l,k}}\left( x \right) = {{\left| {\det A} \right|}^{j/2}}\psi \left( {{B^l}{A^j}x - k} \right)} \hfill \cr {:j,l \in { Z},\;k \in {{ Z}^2}} \hfill \cr } } \right\}$$

where $\psi \in {L^2}\left( {{{ R}^2}} \right)$, that is, the original function represented by *ψ*, which belongs to the function in the two-dimensional square integrable space ${L^2}\left( {{{ R}^2}} \right)$. *j*, *l* and *k* are scale, shear and translation parameters, *A* and *B* are 2 × 2 invertible matrices, which represent the anisotropic expansion matrix of multi-scale partition and the shear matrix for directional analysis, respectively, and satisfy $\left| {\det B} \right| = 1$. When the anisotropic expansion matrix satisfies $A = {A_0} = \left( {\matrix{ 4 & 0 \cr 0 & 2 \cr } } \right)$ and the shear matrix satisfies $B = {B_0} = \left( {\matrix{ 1  1 \cr 0  1\cr } } \right)$, the above equation is called shearlet. For each $\left( {{\xi _1},{\xi _2}} \right) \in {{ D}_0}$, there is

(2) $$\matrix{ {\sum\limits_{j \ge 0} \sum\limits_{l = - {2^j}}^{{2^j} - 1} {{\left| {{{\hat \psi }^{\left( 0 \right)}}\left( {\xi A_0^{ - j}B_0^{ - l}} \right)} \right|}^2} = } \hfill \cr {\sum\limits_{j \ge 0} \sum\limits_{l = - {2^j}}^{{2^j} - 1} {{\left| {{{\hat \psi }_1}\left( {{2^{ - 2j}}{\xi _1}} \right)} \right|}^2}{{\left| {{{\hat \psi }_2}\left( {{2^j}\displaystyle{{{\xi _2}} \over {{\xi _1}}} - l} \right)} \right|}^2}{\rm = }1} \hfill \cr }$$

**Figure 2:**
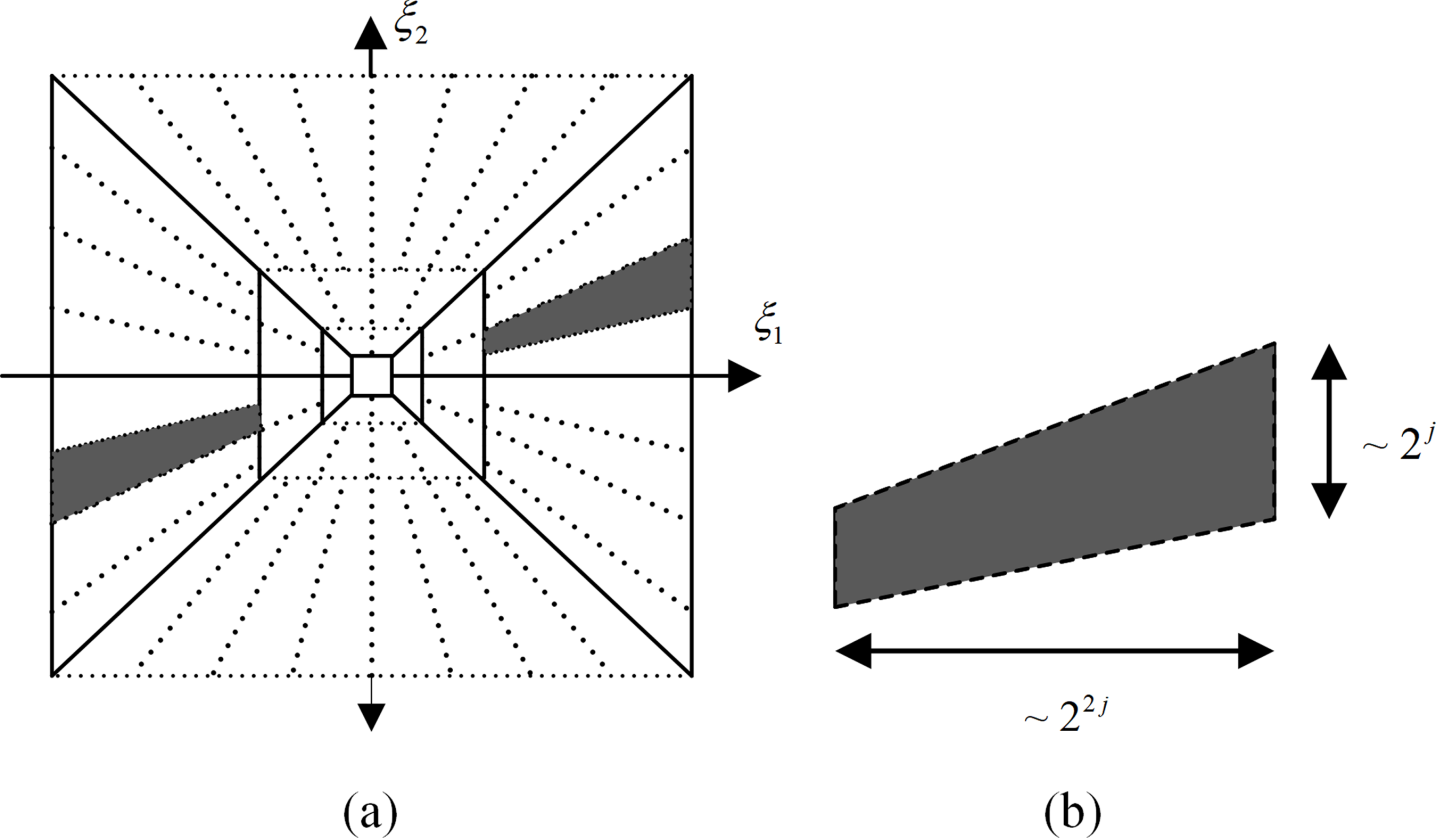
Spatial-frequency plane and frequency support of shearlet: (A) frequency domain split of shearlet, (B) frequency domain support of shearlet.

Where $\hat \psi$ is the Fourier transform result of *ψ*, *ξ*_1_ and *ξ*_2_ are two continuous wavelet functions, and ${\xi _1},{\xi _2} \in {C^\infty }\left( R \right)$. Then $\hat \psi$ satisfies the following formula:

(3) $$\sum\limits_{j{\rm \geqslant }0} \sum\limits_{l = - {2^j}}^{{2^j} - 1} {\left| {{{\hat \psi }^{\left( 0 \right)}}\left( {\xi A_0^{ - j}B_0^{ - l}} \right)} \right|^2} = \sum\limits_{j{\rm \geqslant }0} \sum\limits_{l = - {2^j}}^{{2^j} - 1} {\left| {{{\hat \psi }_1}\left( {{2^{ - 2j}}{\xi _1}} \right)} \right|^2}{\left| {{{\hat \psi }_2}\left( {{2^j}\displaystyle{{{\xi _2}} \over {{\xi _1}}} - l} \right)} \right|^2} = 1$$

where ${{ D}_0} = \left\{ {\left( {{\xi _1},{\xi _2}} \right) \in {{\hat { R}}^2}:\left| {{\xi _1}} \right| \ge 1/8,\;\left| {{\xi _2}/{\xi _1}} \right| \le 1} \right\}$. The function $\left\{ {{{\hat \psi }^{\left( 0 \right)}}\left( {\xi A_0^{ - j}B_0^{ - l}} \right)} \right\}$ is a split of ***D***_0_, which is shown in Fig. 2A. Because the set $\left\{ {\matrix{ {\psi _{j,l,k}^{\left( 0 \right)}\left( x \right){{ = 2}^{{{3j} \over 2}}}{\psi ^{\left( 0 \right)}}\left( {B_0^lA_0^jx - k} \right)} \hfill \cr {:j \ge 0,\; - {2^j} \le l \le {2^j} - 1,\;k \in {{ Z}^2}} \hfill \cr } } \right\}$ is a Parseval frame of ${L^2}{\left( {{{ D}_0}} \right)^{^ \vee }} = \left\{ {f \in {L^2}\left( {{{ R}^2}} \right):{\rm supp}\ \hat f \subset {{ D}_0}} \right\}$. It is easy to get the support of function *ψ*_*j*,*l*,*k*_ in the frequency domain from the support set of functions ${\hat \psi _1}$ and ${\hat \psi _2}$ (Lim, 2010).

(4) $$\mathop {{\rm s}upp}\nolimits_ {\kern 1pt} \hat \psi _{j,l,k}^{(0)} \subset \left\{ {\matrix{ {\left( {{\xi _1},{\xi _2}} \right):} \hfill \cr {{\xi _1} \in \left[ { - {2^{2j - 1}}, - {2^{2j - 4}}} \right] \cup \left[ {{2^{2j - 4}}{{,2}^{2j - 1}}} \right]} \hfill \cr {,\left| {{\xi _2}/{\xi _1} + l{2^{ - j}}} \right| \le {2^{ - j}}} \hfill \cr } } \right\}$$

It can be obtained from the above formula that each element ${\hat \psi _{j,l,k}}$ is supported on a trapezoidal pair with a slope of *l*2^−*j*^ and an approximate size of 2^2*j*^ × 2^*j*^, as shown in Fig. 2B. In the spatial domain, the shearlet has a fast attenuation characteristic, and the scale coefficient in any direction can be obtained by adjusting the translation parameters; at the same time, in the frequency domain, the shearlet transform is compact support. Therefore, it can be concluded that shearlets have good local characteristics. Since the supporting base of each element is along the *l*2^−*j*^ direction, and the number of shearlet transform directions will increase layer by layer with the continuous refinement of the scale, the shearlet has strong directional sensitivity.

In addition to the above features, shearlet also have an optimal sparse representation. Denoting *f* as the function that the direction is *C*^2^ except a curve that is piecewise *C*^2^ continuous respectively. In the expansion $f = \sum\nolimits_{j,l \in Z,\;k \in {Z^2}} \left\langle {f,{\psi _{j,l,k}}} \right\rangle {\psi _{j,l,k}}$, the result of approximating the function *f* with the maximum shearlet coefficient of the *N* term is $f_N^S$ (De Oliveira, Carvalho-Ribeiro & Maia-Barbosa, 2020). The relationship between them is

(5) $$\left\| {f - f_N^s} \right\|_2^2\ \  \le C{N^{ - 2}}{\left( {\log N} \right)^3}$$

Obviously, the approximation order of the shearlet is $O\left( {{N^{ - 2}}{{\left( {\log N} \right)}^3}} \right)$, and its approximation accuracy is much higher than that of the traditional wavelet (its nonlinear error approximation order is $O\left( {{N^{ - 1}}} \right)$), and it is already very close to the theoretically optimal nonlinear error approximation order $O\left( {{N^{ - 2}}} \right)$. This shows that the shearlet can optimally represent images with edges in various directions and scales.

## Methods

Aiming at the shortcomings of low detection accuracy and insensitive direction of the existing bankline detection algorithms, we hope to design a bankline detection algorithm suitable for GF-3 SAR images. Due to the special imaging mechanism of GF-3 SAR image, the difficulty of the algorithm is not only to overcome speckle the interference of SAR image, but also to ensure the accuracy and smoothness of the detection results. Therefore, we combine the advantages of nonlocal mean filtering, shearlet and morphological processing to construct a joint algorithm. Firstly, the non-local mean filter is used to preprocess the GF-3 SAR image in this algorithm, so as to reduce the interference of speckle on bankline detection. Secondly, the logarithmic transformation is used to turn the multiplicative speckle model of GF-3 SAR image into additive, and then the shearlet is used to detect the bankline of the image. Then, exponential transformation is performed on the detection result, and the image is binarized using the OTSU algorithm, and the hit-or-miss transformation is used to refine the bankline. Finally, aiming at the problem of a small amount of false banklines in this result, the false banklines were removed to obtain the final result. Because the proposed algorithm uses non-local mean filter to preprocess the image, the speckle interference in the detection results is weaker; the shearlet is used to analyze the image, so the detected bankline position is more accurate; the morphology is used to optimize the results, so the detected bankline is smoother.

### Preprocessing of non-local mean filtering

In order to get a complete and accurate result of bankline detection, it is necessary to select an appropriate filtering method to preprocess the image, so as to suppress the interference of speckle in GF-3 SAR image and effectively retain the bankline information that needs to be detected. Non-local mean filtering algorithm (Isola et al., 2017; Buades, Coll & Morel, 2005) uses structural similarity to define the distance between pixels, and makes full use of various redundant information possessed by the image itself, which can better protect the edge and texture features of the image. Therefore, in this section, the non-local mean filter is selected to preprocess the GF-3 SAR image, and a to-be-processed image is obtained, in which the speckle is suppressed effectively and the bankline information is preserved effectively.

For a given GF-3 SAR image $v = v\left\{ {v\left( i \right)|i \in I} \right\}$, *I* represents a bounded image domain. For a certain pixel *i* in the image, the estimated value after non-local mean filtering is shown as Eq. (6).

(6) $$N|L[v](i) = \sum\limits_{j \in I} w(i,j)v(j)$$

Where, *N*|*L*[*v*](*i*) is the preprocessing result of non-local mean filtering, *w*(*i*,*j*) is the noise reduction block, *i* and *j* are two pixels, and pixel *j* is any pixel in the image domain *I* except pixel *i*. The weight *w*(*i*,*j*) depends on the similarity between the pixel *i* and the pixel *j*, and satisfies the conditions 0 *≤ w*(*i*,*j*) *≤* 1 and $\sum\nolimits_j w(i,j) = 1$. It can be seen from the above formula that the algorithm uses a fixed size image block to match in the whole image, selects the image block with large similarity to this image block, and then gives the corresponding weight to the image blocks to perform filtering based on the similarity.

### Logarithmic transformation

The imaging process of SAR images can be regarded as a small-aperture antenna with a constant speed moving on a long linear array orbit and sending detection waves, and then coherently processing the corresponding echoes received in different orbital regions, so as to achieve the imaging effect of a large-aperture radar. Due to the existence of electromagnetic interference, the image will appear speckle. These speckle obeys the following multiplicative model (Sun & Han, 2010):

(7) $$Y = FX$$

where *Y* is an imaging result, *F* is speckle, and *X* is a real result.

Since the additive model can give full play to the advantages of shearlet, it is necessary to change the multiplicative model into additive model to optimize the detection process.

(8) $${\rm log}\left( Y \right) = {\rm log}\left( {F} \right) + {\rm log}\left( {X} \right)$$

### Bankline detection

The classical definition of shearlet system is shown in Eq. (1). According to the definition, shearlets can be aligned vertically in the time domain, and the perfect horizontal alignment can be achieved by increasing the shearlet parameters to infinity. However, this operation will lead to extremely low application of shearlet transform in the vertical direction, which will bring great difficulties to practical applications. Based on this, we consider using the complex shearlet transform to satisfy the symmetry and translation invariance of the time domain and frequency domain. The definition of complex shearlet is defined as:

(9) $$\psi = {\psi ^{even}} + i{\psi ^{odd}} = {\psi ^{even}} + iH\left( {{\psi ^{even}}} \right)$$

where *ψ*^*even*^ is even symmetric shearlet and *ψ*^*odd*^ is odd symmetric shearlet. Since the classic shearlet is symmetric about *y* , *ψ*^*even*^ has been obtained. *ψ*^*odd*^ can be obtained from *ψ*^*even*^ according to the Hilbert transformation (Xu et al., 2020), and the transformation formula is defined as:

(10) $$H(f)(t) = \mathop {\lim }\limits_{a - \gt \infty } \int_{ - a}^a \displaystyle{{f(\tau )} \over {t - \tau }}d\tau$$

The definition of even symmetric shearlet and odd symmetric shearlet is defined as:

(11) $$\psi _{j,k,x}^{e{\rm ven}}( \cdot {) = 2^{{{3j} \over 2}}}{\psi ^{even}}({S_k}{A_j}( \cdot - x))$$

(12) $$\psi _{j,k,x}^{odd}( \cdot {) = 2^{{{3j} \over 2}}}{\psi ^{odd}}({S_k}{A_j}( \cdot - x))$$

where *j* ∈ *N*_0_, $\left| k \right| < \left\lceil {{2^{{j \over 2}}}} \right\rceil$ and *x* ∈ *Z*^2^ are scale, shear and translation parameters, ${S_k} = \left( {\matrix{ 1 & k \cr 0 & 1 \cr } } \right)$ is the shear matrix and ${A_j} = \left( {\matrix{ {{2^j}} & 0 \cr 0 & {{2^{j/2}}} \cr } } \right)$ is the anisotropic expansion matrix.

A simple and intuitive way to calculate the likelihood of the presence of banklines in a GF-3 SAR image is to maximize all possible directions. However, experiments have shown that a more satisfactory result can be achieved by pre-selecting the main direction of the bankline. The primary direction can be determined by the maximum coefficient associated with the odd symmetric shearlet. That is to say, for each point in the two-dimensional plane, we will select the value with the largest absolute value among all the coefficients related to the odd symmetric shearlet, and select the corresponding shearlet direction as the main direction. Finally, based on the selected direction, the even symmetric shearlet coefficient of the image can be calculated by using Eq. (11), and the possibility of bankline on the pixel can be calculated by combining the odd symmetric shearlet coefficient obtained by Eq. (12).

For the scale parameter *j* ∈ {*J*_min_,…,*J*_max_}, the shear parameter $\left| k \right| \le \lceil {{2^{{j \over 2}}}} \rceil$ and the translation parameter *x* ∈*R*^2^, the shearlet direction is taken as the optimal direction for the bankline by selecting the maximum of the absolute values of all the coefficients. The optimal direction $k_{{\psi ^{even}},{\psi ^{odd}}}^ * \left( {f,x} \right) \in \left\{ { - {2^{{{{J_{\min }}} \over 2}}},....,{2^{{{{J_{\max }}} \over 2}}}} \right\}$ of the bankline at each pixel point *x* ∈ *R*^2^ is calculated by:

(13) $$k_{{\psi ^{even}},{\psi ^{odd}}}^*\left( {f,x} \right) = \mathop {\arg \max }\limits_{\tilde{k} \in \left\{ { - \left\lceil {{2^{{{{J_{\min }}} \over 2}}}} \right\rceil , \ldots .,\left\lceil {{2^{{{{J_{\max }}} \over 2}}}} \right\rceil } \right\}} \mathop {\max }\limits_{\matrix{
   {}  \cr 
   {j \in \left\{ {{J_{\min ,}}, \ldots ,{J_{\max }}} \right\},}  \cr 
   {k \in \left\{ { - \left\lceil {{2^{{j \over 2}}}} \right\rceil , \ldots ,\left\lceil {{2^{{j \over 2}}}} \right\rceil } \right\},}  \cr 
   {\left| {\tilde{k} - k\left\lceil {{2^{{{{J_{\min }}} \over 2}}}} \right\rceil {{\left\lceil {{2^{{j \over 2}}}} \right\rceil }^{ - 1}}} \right|{\kern 1pt}  \le {\kern 1pt} {1 \over 2}{\rm{ }}}  \cr 

 } } \left| {\left. {f,\left\langle {\psi _{j,k,x}^{odd}} \right.} \right\rangle } \right|$$

where $\left\langle {f,{\psi _{j,k}}} \right\rangle$ is used to represent the coefficient calculated at the translation parameter *x* of the shearlet, and the maximum value is obtained for any scale parameter *j* > 0 and $\left\langle {f,\psi _{j,0}^{odd}} \right\rangle$, and $\left\langle {f,\psi _{j,0}^{even}} \right\rangle =0$ is satisfied. Then the probability of whether each pixel *x* ∈ *R*^2^ is a point on the bankline is calculated by the following formula:

(14) $${\tilde E_{_{{\psi ^{even}},{\psi ^{odd}}}}}(f,x) = \displaystyle{{\left| {\sum\limits_{j\ =\ {J_{\min }}}^{{J_{\max }}} \langle f,\psi _{j,{k_j},x}^{odd}\rangle } \right| - \sum\limits_{j\ =\ {J_{\min }}}^{j\ =\ {J_{\max }}} \left| {\langle f,\psi _{j,{k_j},x}^{even}\rangle } \right|} \over {\left( {{J_{\max }} - {J_{\min }} + 1} \right)\mathop {\max }\limits_{j\  \in\  \left\{ {{J_{\min }},\ldots,{J_{\max }}} \right\}} \left| {\langle f,\psi _{j,{k_j},x}^{odd}\rangle } \right| + \varepsilon }}$$

where *f* is the image matrix, ${S_k} = \left( {\matrix{ 1  k \cr 0  1 \cr } } \right)$ is the shear matrix, and ${A_j} = \left( {\matrix{ {{2^j}} & 0\cr 0 & {{2^{j/2}}} \cr } } \right)$ is the anisotropic expansion matrix. $\varepsilon \left( {\varepsilon > 0} \right)$ in the above equation is the minimum value approaching zero to ensure that the denominator will not be zero. When the shearlet takes the ideal bankline as the center of symmetry, the following equation is satisfied:

(15) $$\matrix{
   {{{\left| {\sum\limits_{j\; = \;{J_{\min }}}^{{J_{\max }}} {\langle f,\psi _{j,{k_j},x}^{odd}\rangle } } \right| - \sum\limits_{j\; = \;{J_{\min }}}^{j\; = \;{J_{\max }}} {\left| {\langle f,\psi _{j,{k_j},x}^{even}\rangle } \right|} } \over {\left( {{J_{\max }} - {J_{\min }} + 1} \right)\mathop {\max }\limits_{j \in \left\{ {{J_{\min }}, \ldots ,{J_{\max }}} \right\}} \left| {\langle f,\psi _{j,{k_j},x}^{odd}\rangle } \right| + \varepsilon }}}  \cr 
   {{\rm{ = }}{{\left| {\sum\limits_{j\; = \;{J_{\min }}}^{{J_{\max }}} {\langle f,\psi _{j,{k_j},x}^{odd}\rangle } } \right|} \over {I \cdot \mathop {\max }\limits_{i \in \left\{ {1,2,3, \ldots ,I} \right\}} \left| {\left\langle {f,\psi _{{j_i},x}^{odd}} \right\rangle } \right|}}{\rm{ = }}1}  \cr 

 } $$

So $0 < = {\tilde E_{_{{\psi ^{even}},{\psi ^{odd}}}}}\left( {f,x} \right) < = 1$ is the probability that *x* is the bankline. In order to make the bankline measurement probability value of each pixel between 0 and 1, we set the following mapping relationship finally (Sun et al., 2020):

(16) $${E_{_{{\psi ^{even}},{\psi ^{odd}}}}}(f,x) = \max ({\tilde E_{_{{\psi ^{even}},{\psi ^{odd}}}}}(f,x),0)$$

The mapping relationship generates a value between 0 and 1, which is used to represent the existence probability of the bankline at a certain position of the measurement. In other words, a metric of 0 means that there is no bankline at the location, and 1 means that there must be a bankline.

### Binarization and refinement of bankline

Image binarization is the process of transforming a multi-gray-level image into a two-gray-level image through a certain processing method. The threshold binarization algorithm is to calculate a threshold *T*(*x*,*y*) for an *M* × *N* pixel image *I*(*x*,*y*), where *x* and *y* are the two-dimensional coordinate positions in the image. Then when *I*(*x*,*y*) *≤ T*(*x*,*y*), the corresponding pixels of binary image satisfy *B*(*x*,*y*) = 0; on the contrary, the corresponding pixels of binary image satisfy *B*(*x*,*y*) = 255. Currently commonly used overall threshold binarization methods include gray-scale expected value method (Pierre et al., 2017), OTSU method (Khambampati et al., 2018), maximum variance ratio method (Liu, Wang & Yu, 2013), etc., most of which are automatically acquired according to the gray-level distribution characteristics of specific images, that is, they are adaptive, and their principles have a solid theoretical basis.

Because the result of using shearlet to detect the bankline is a probability value between 0 and 1, the OTSU algorithm with better stability is selected to calculate the global threshold when binarizing the result. In the OTSU algorithm (Khambampati et al., 2018), a gray value *T* is selected to segment the image into two parts: foreground and background. We call one of them *A* and the other *B*. Note that *E*_*all*_ is the overall expectation of the image, *E*_*A*_ is the expectation of *A*, *E*_*B*_ is the expectation of *B*, *P*_*A*_ is the probability of class *A*, *P*_*B*_ is the probability of class *B*, and the probability of each gray value is *pi*, and *σ*^2^ is the variance between classes. The following equation is satisfied:

(17) $${E_{all}} = \sum\limits_{i = 0}^{255} i*pi$$

(18) $$Su{m_A} = \sum\limits_{i = 0}^T i*pi$$

(19) $${P_A} = \sum\limits_{i = 0}^T pi$$

(20) $${E_A} = \displaystyle{{Su{m_A}} \over {{P_A}}}$$

(21) $$Su{m_B} = \sum\limits_{i = T}^{255} i*pi$$

(22) $${P_B} = 1 - {P_A}$$

(23) $${E_B} = \displaystyle{{Su{m_B}} \over {{P_B}}}$$

(24) $${\sigma ^2} = {P_A}*({E_{all}} - {E_A}{)^2} + {P_B}*({E_{all}} - {E_B}{)^2}$$

When the gray value *T* makes the inter class variance *σ*^2^ maximum, then *T* is the threshold obtained by OTSU method. According to the threshold value, each pixel in the image is traversed. If the pixel value is greater than or equal to the threshold value, the gray value of the point is set to 1. If the pixel value is less than the threshold value, the gray value of the point is set to 0. The result image of bankline detection is converted into a binary image.

The refinement of the bankline requires the use of mathematical morphology tools (Ghamisi et al., 2018) to process the binary image. Mathematical morphology is developed based on set theory. Its main purpose is to simplify the image, remove useless information, and retain the required image structure. An important operation in morphological processing is refinement (Sun et al., 2020), which uses the detection result of the hit-or-miss transformation (Sakamat, Abd Khalid & Azha, 2018) to continuously remove some points along both sides of the image edge until the edge width reaches a single pixel. The principle of thinning by hit-or-miss transform is to extract the pixel regions in the image that match the given neighborhood structure by hit-or-miss transform, and then subtract these pixel regions from the original image to get the image skeleton with only one pixel width.

When using hit miss transformation, we need to construct structure element $b\left( {x,y} \right)$ first. The structure element is composed of two disjoint parts ${b_1}\left( {x,y} \right)$ and ${b_2}\left( {x,y} \right)$, which are used to match on both sides of the edge. Then, the definition of the matching between the input image $f\left( {x,y} \right)$ and the structural element $b\left( {x,y} \right)$ is as follows:

(25) $$f*b\left( {x,y} \right) = \left( {f{\rm \odot }{b_1}} \right) \cap \left( {{f^c}{\rm \odot }{b_2}} \right)$$

where * represents the matching operation, :amp:odot; represents the corrosion operation, and *f*^*c*^ represents the complement of *f*. Further, we can know that the definition of bankline refinement by using the hit-or-miss transform is as follow, where :amp:otimes; represents the refinement operation:

(26) $$f \otimes b\left( {x,y} \right) = f - f*b\left( {x,y} \right)$$

Due to the existence of the burr phenomenon of the bankline, the skeleton shift phenomenon will occur in some positions as a result of the hit-or-miss transformation. In order to make the detection result of the bankline more accurate, we need to deal with the burr phenomenon of the bankline before the bankline is refined. The specific method is to first expand the image to make the bankline of the image smooth and then perform the refinement operation.

In a two-dimensional Euclidean space *Z*^2^, let *f*(*x*,*y*) be the input image, *b*(*s*,*t*) be the structural element, and use *b*(*s*,*t*) to expand the input image *f*(*x*,*y*) as follows. Among them, *D*_*f*_ is the spatial domain of the input image, and *D*_*b*_ i s the spatial domain of the structural element.

(27) $$f \oplus b\left( {x,y} \right) = \max \left\{ {\matrix{ {f\left( {x - s,y - t} \right) + b\left( {s,t} \right)|\left( {x - s} \right),} \hfill \cr {\left( {y - t} \right) \in {D_f},\left( {s,t} \right) \in {D_b}} \hfill \cr } } \right\}$$

The above two-step morphological processing principle is simple and easy to implement, and the bankline can be refined and a large number of false bankline can be removed, and finally obtain a clear binary image of the bankline.

### False bankline elimination

In the previous section, the detection result of bankline has been converted into binary image, so the first step of removing the false bankline is to mark the connected region in binary image. Marking the connected domain in the binary image, that is, in an image composed of white pixels (usually represented by “255” in the grayscale image and by “1” in the binary image) and black pixels (usually represented by “0”), the pixel sets having pixel values “1” or “255” adjacent to each other are marked with different numbers. And the number of white pixels is counted in different connected domains (Liu et al., 2013).

In GF-3 SAR images, the false bankline formed by speckle generated by the coherent imaging mechanism are usually small connected areas. The threshold is set, and the portion where the number of pixels in each connected region of the image is less than the threshold is assumed to be a false bankline. The pixel values in these connected regions are all set to 0 to achieve the purpose of eliminating false banklines.

### Overall detection steps

**Figure 3:**
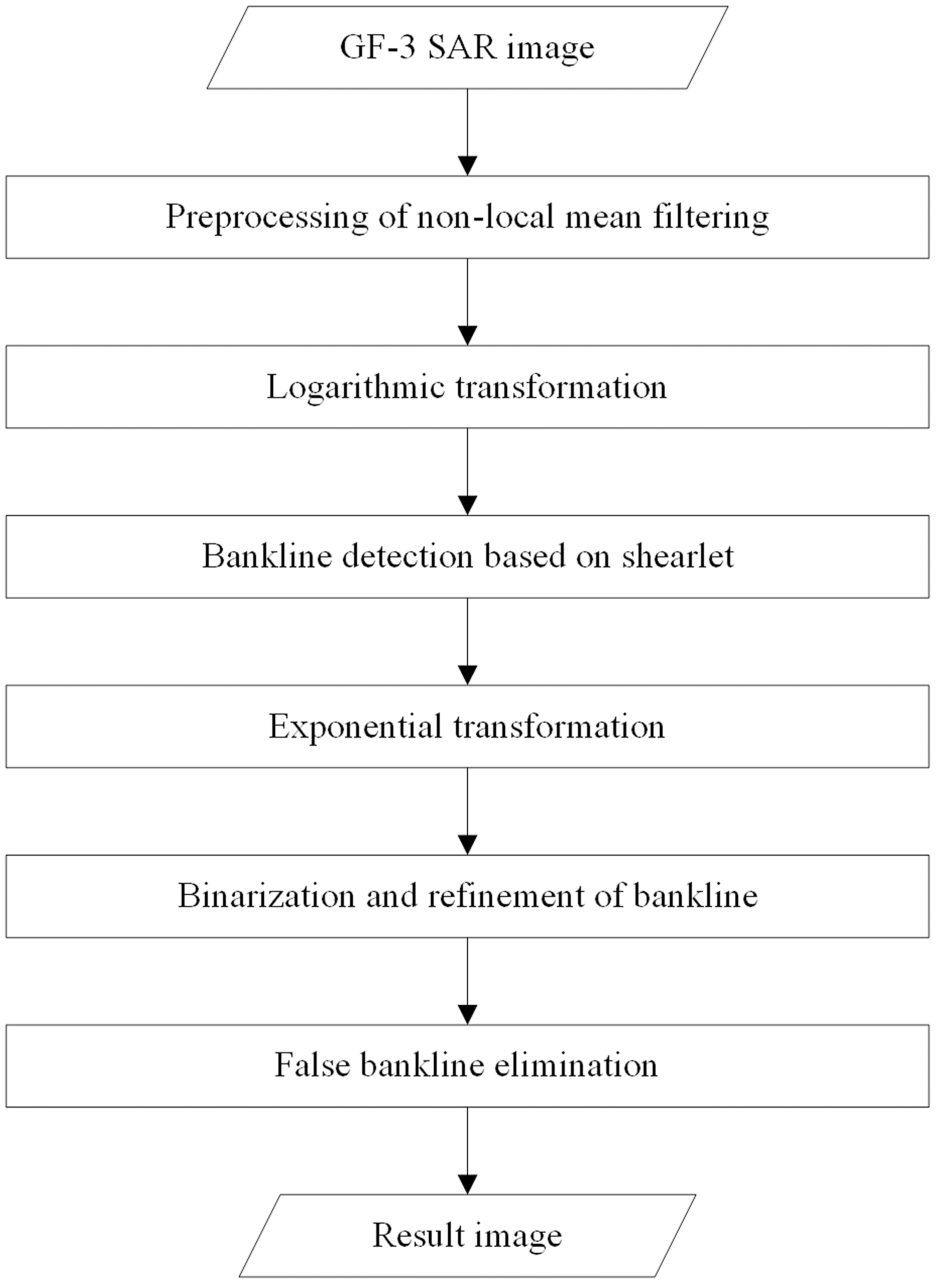
Flow chart of bankline detection.

In a word, the flow chart of bankline detection in GF-3 SAR image presented in this paper is shown in Fig. 3. Firstly, aiming at the speckle of GF-3 SAR image, we use non-local mean filtering to preprocess the GF-3 SAR image, in order to reduce the influence of speckle on the accuracy of bankline positioning. Secondly, the logarithmic transformation is used to turn the multiplicative speckle model into additive, which facilitates the detection of bankline information using shearlet. Then, even symmetric shearlet and odd symmetric shearlet are used to transform the image to generate coefficients, and a series of calculations are performed on the coefficients to calculate the probability of a bankline on the pixel for each pixel in the image. Since the shearlet has optimal sparsity and strong directionality, the position and direction of the bankline in the result have high accuracy. Then, exponential transformation is performed on the detection result, and the OTSU algorithm is used to select an appropriate threshold to binarize the image, and the bankline is thinned by the hit-or-miss transformation. Finally, aiming at the problem of a small amount of false banklines in the result, the false banklines were removed, the results were optimized, and the final result was obtained.

## Results

In this section, we use three GF-3 SAR images taken near inland cities to verify the proposed algorithm. That is to say, this algorithm is suitable for bankline detection in inland areas.

**Figure 4:**
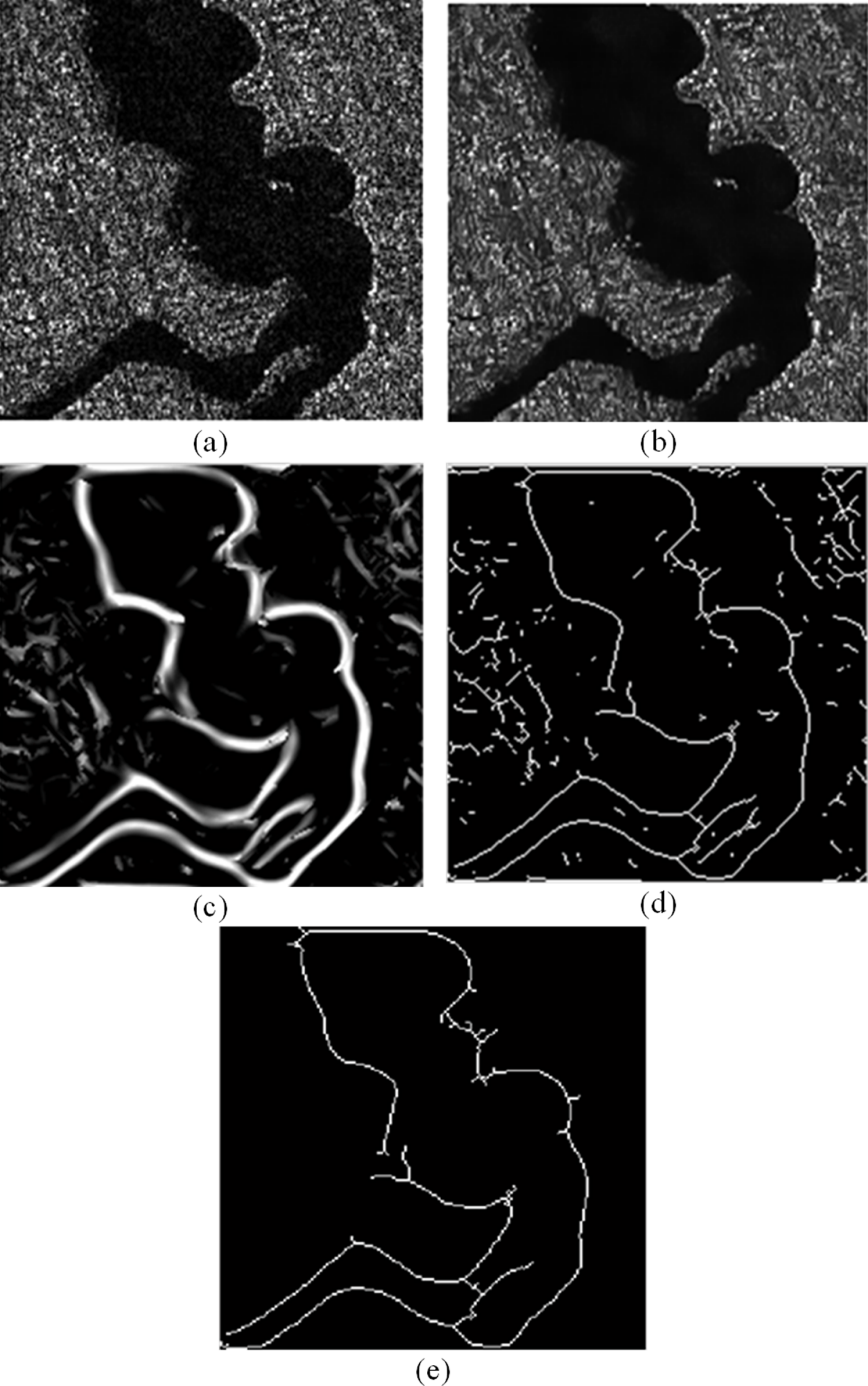
The detection results of the proposed method: (A) original image (B) non-local mean filtering preprocessing result (C) shearlet detection result (D) binarization and refinement result (E) final result.

We set the block size for calculate weight in the non-local mean filter to 2; set the maximum scale parameter of the complex shearlet to 2, and the minimum scale parameter to 6; set the refinement parameter to 0.2, the bankline detection result of a GF-3 SAR image as shown in Fig. 4 is obtained. Fig. 4A is selected from GF-3 SAR image generated by HHHV polarization in fine strip-2 imaging mode on November 21, 2017. The longitude and latitude of the center of the image are 108°1′E and 33°9′N, with a resolution of 10 m. Obviously, non-local mean filter preprocessing can effectively reduce speckle interference and effectively retain the information of the bankline to be detected. Next, the use of shearlets with optimal sparsity and strong directionality can effectively detect the bankline information in the image, ensuring the detection accuracy and continuity of the bankline. Then, the OTSU algorithm is used to binarize the image, and the hit-or-miss transformation is used to refine the bankline. Finally, the false bankline caused by a small amount of speckle interference is removed by means of statistical connected domains, and the final bankline detection result is obtained. In a word, due to the optimal sparsity and strong directionality of shearlet, the bankline information of the GF-3 SAR image which also has obvious sparse characteristics can be effectively detected.

**Figure 5:**
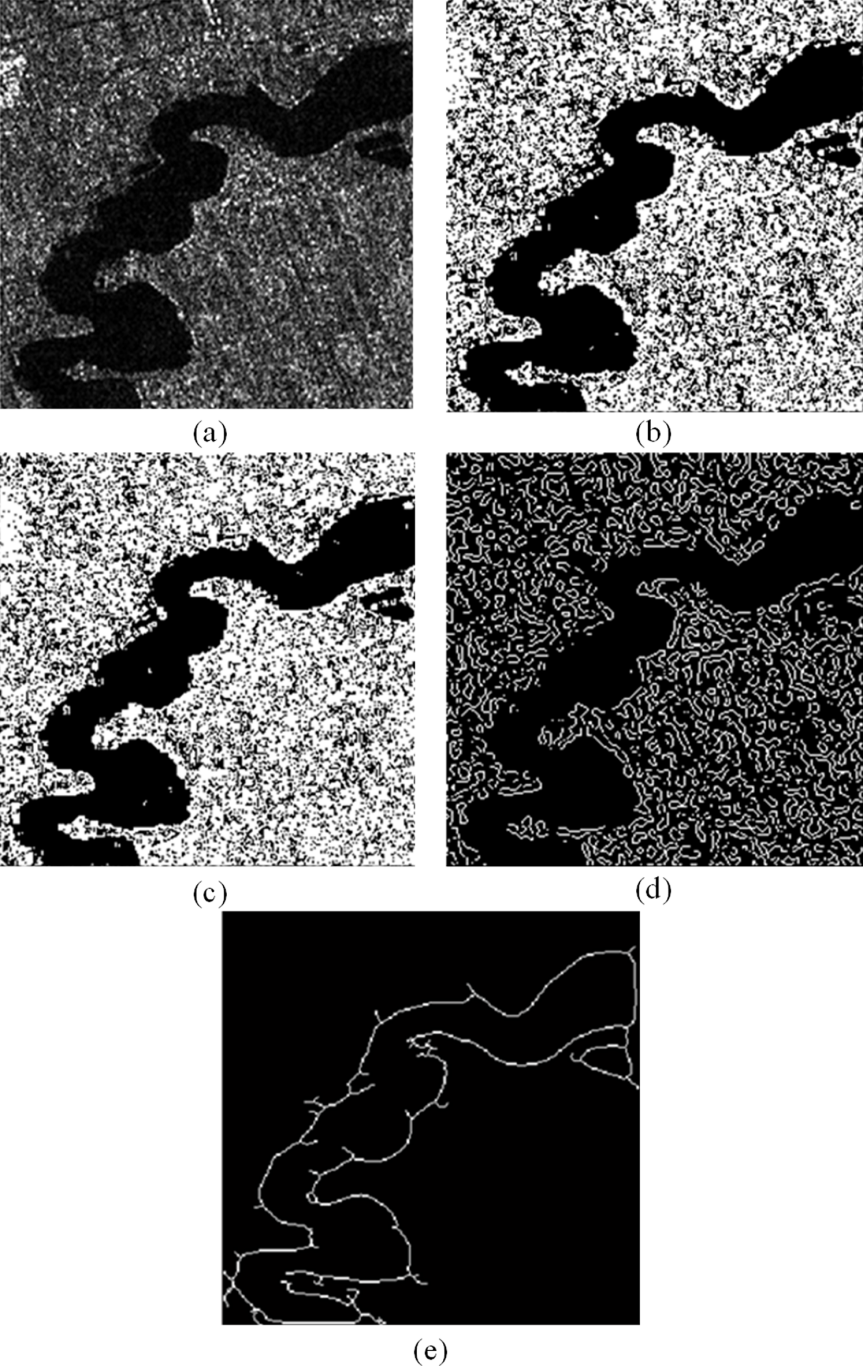
Bankline detection results of GF-3 SAR image with different methods: (A) original image (B) Sobel detection (C) Prewitt detection (D) Log detection (E) proposed detector.

Figure 5 shows the detection results of the bankline in another GF-3 SAR image, the bankline is thinned by the hit-or-miss transformation. The imaging parameters of the original image are shown in Table 1. The Sobel operator and the Prewitt operator are differential template operators that use a template to convolve the image and extract edges. The difference between the two is that the Sobel element has a corresponding weighting in the 4-neighbor position of the pixel, so the Sobel operator suppresses the speckle ability stronger than the Prewitt operator, but the detection edge is wider. Log operator is composed of two parts. Firstly, Gaussian convolution function is used to filter the image, and then Laplacian operator is used to detect the edge of the image. Therefore, the Log operator has a good suppression effect on noise and discrete points in image.

Obviously, the Sobel operator and Prewitt operator cannot overcome the effects of speckle in the GF-3 SAR image, and combine the real bankline with the false bankline, and the bankline detection results are chaotic. In the detection result of the Log operator, the bankline appears serious rupture phenomenon, but the trend of the river can be barely seen as a whole. These three traditional detection operators are all based on difference, so the interference of the speckle is extremely serious, which is not suitable for the bankline detection of the GF-3 SAR image. The proposed edge detection makes full use of optimal sparsity and strong directionality of shearlet. It uses non-local mean preprocessing, shearlet bankline detection, and morphological processing in sequence. Non-local mean preprocessing can suppress the speckle of GF-3 SAR image sufficiently, and retain bankline information effectively. Shearlet can detect possible banklines in multiple directions in a multi-dimensional space, and the detected bankline positions are more accurate than other multi-scale geometric analysis tools. At the same time, morphological processing, including false bankline removal, bankline burr removal and bankline refinement operations, is performed to optimize the detection results to obtain ideal bankline detection results. In a word, the accuracy of the bankline detection results of this algorithm is higher, and the bankline is more complete and smooth, which is very suitable for the detection of bankline information under GF-3 SAR image speckle interference.

The proposed method combines the non-local mean filtering, shearlet detection, OTSU algorithm and morphological processing, which uses the non-local mean filtering to suppress the speckle, takes advantage of the optimal sparsity and strong directionality of shearlet, and uses the OTSU algorithm and morphological processing to binarize and refine bankline, so the ideal detection result can be finally obtained. In a word, the experimental results show that the accuracy of the bankline detection result of the proposed algorithm is higher, and the bankline is more complete and smooth, which is very suitable for the detection of bankline information under interference of strong speckle and complex background in the GF-3 SAR image.

Table 3 shows the qualitative evaluation results of the proposed algorithm and Sobel, Prewitt and Log. In the table, four indicators are used to evaluate the algorithm, which are anti-speckle, smoothness, completeness and running speed. The anti-speckle indicator is used to measure the ability of the algorithm to overcome the effect of speckle, which is of great significance to the detection result of bankline under strong speckle interference. The smoothness indicator is used to measure the continuous and unbreakable degree of the bankline in the test result. Completeness indicator is used to measure the clarity of direction and outline of the bankline in the detection result. The running speed indicator is used to measure how fast the algorithm runs. For the anti-speckle ability of the detection result, since the Sobel operator and Prewitt operator have no noise reduction ability, the anti-speckle ability is weak; since the Log operator uses a preliminary low-pass filter, the anti-speckle ability is medium; Our algorithm uses an advanced noise reduction function, which can achieve an effective balance between noise suppression and information retention, so the anti-speckle ability is strong. For the smoothness of the detection results, the detection results of Sobel, Prewitt and Log operator are seriously split by the speckle, so the smoothness is low; since our algorithm can overcome the interference of speckle, the smoothness of the detected bankline is high. For the completeness of the detection results, since the Log operator is severely interfered by the speckle, the bankline is completely split, so the completeness is low; since the detection results of the Sobel operator and the Prewitt operator can barely identify the contour and trend of the bankline, but the real bankline and the false bankline cannot be distinguished, so the completeness is medium; our algorithm can overcome the interference of speckle, the detection result of the bankline is not obviously split, so the completeness is high. Regarding the running speed of the algorithm, it can be seen that the running time of the Sobel operator, Prewitt operator and Log operator when processing the same image is less than 1 s, so the running speed is fast; our algorithm is a combination of many methods, and the cumulative running time is more than 30 s, so the running speed is slow.

**Table 3:** Imaging parameters of GF-3 SAR image.

Detectors	Anti-speckle	Smoothness	Completeness	Running speed
Sobel	Weak	Low	Medium	Fast
Prewitt	Weak	Low	Medium	Fast
Log	Medium	Low	Low	Fast
Our algorithm	Strong	High	High	Slow

**Figure 6:**
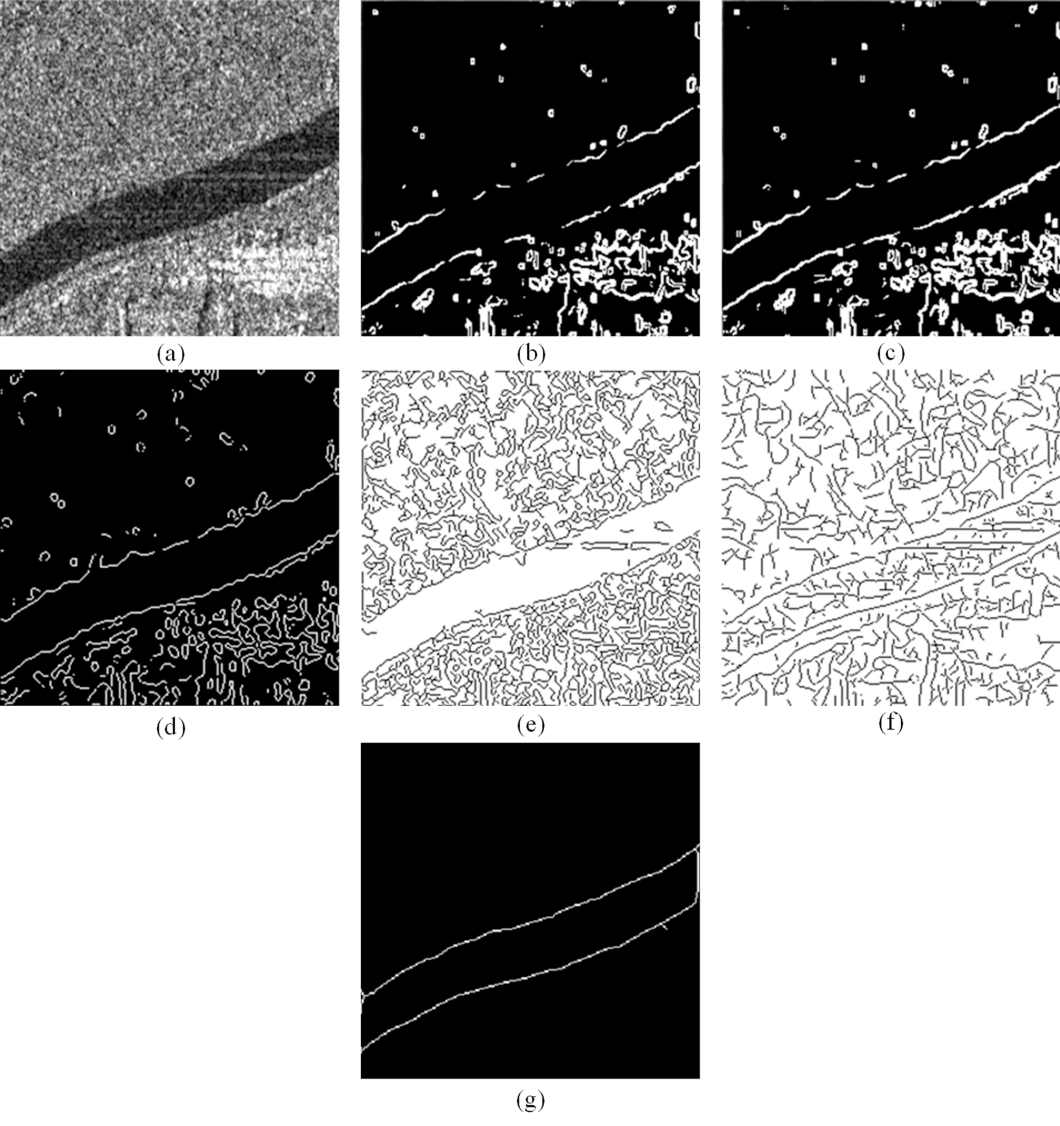
Bankline detection results of different methods: (A) original image (B) Sobel detection (C) Prewitt detection (D) Log detection (E) algorithm in Fuping & Penglang (2016), (F) algorithm in Penglang & Dong (2012) (G) proposed algorithm.

Figure 6A is selected from the SAR image generated by GF-3 satellite in orbit No. 006162 on October 11, 2017, using FS2 mode and HHHV polarization, with the central longitude and latitude of 109°8′E, 34°1′N, with resolution of 10 m. Figure 6 shows three detection results based on difference operator, the detection results of algorithms proposed in Wang & Shui (2016) and Shui & Chen (2012), and the detection result of the proposed algorithm. The algorithm proposed in Wang & Shui (2016) is based on a gradient matrix and an anisotropic Gaussian directional derivative matrix, which can detect edge information in a color image interfered by Gaussian white noise or a small percentage of impulse noise. The algorithm proposed in the paper (Shui & Chen, 2012) uses a Gauss-Gamma-shaped double window to reduce false edge pixels near the real edge, and can extract the fine edge of SAR image more accurately. Similar to the detection results in Fig. 5, Sobel, Prewitt and Log operators cannot overcome the influence of speckle in GF-3 SAR image, and the detection result have obvious cracking phenomenon. Obviously, the detection results of the algorithms in Wang & Shui (2016) and Shui & Chen (2012) are severely disturbed by speckle, and the detected banklines are poor in smoothness and continuity. Since the algorithm in Wang & Shui (2016) can overcome the interference of Gaussian white noise and a small percentage of impulse noise in the color image, the false bankline in the detection result is less than those of the algorithm in Shui & Chen (2012). The river bankline detected by our proposed algorithm has the highest degree of smoothness and continuity among all comparison algorithms. Table 4 shows the difference in running time for the six algorithms. Obviously, the three traditional detection operators use simple templates for detection, so the running time is short. The algorithms in Wang & Shui (2016) and Shui & Chen (2012) use matrix operations and double-window detection respectively, so the running time is slower than that of template detection. Since the proposed algorithm is a combination of many methods, the running time is the longest compared to other five comparison algorithms.

**Table 4:** Running time of different detectors.

Detectors	Sobel	Prewitt	Log	Detector in Wang & Shui (2016)	Detector in Shui & Chen (2012)	The proposed detector
Running speed	0.028	0.085	0.773	7.309	4.358	33.571

In a word, the proposed algorithm uses a combination of non-local mean filtering and morphological processing to overcome the effects of speckle when the bankline information in the GF-3 SAR image is interfered by strong speckle and is difficult to detect. Therefore, the proposed algorithm has a strong anti-speckle ability. Moreover, the smoothness and completeness of the detection results of the proposed algorithm are very high, because the shearlet with optimal sparsity and strong directionality are used to detect bankline information. However, because the proposed algorithm is a combination of multiple methods and many steps, it runs slowly. In a word, the proposed algorithm is very suitable for detecting bankline information under strong speckle interference in GF-3 SAR images, but the operation speed needs to be improved. We further analyze the differences in accuracy of all algorithms. The comparison algorithms are affected by speckle, and the detection results have obvious breaks and discontinuities. The proposed algorithm combines the advantages of several methods, so it can overcome the interference of speckle of GF-3 SAR image, and can detect the complete and clear bankline contour, leading to the highest detection accuracy among all the algorithms.

## Critical discussion

Compared with the existing methods, the proposed algorithm has obvious advantages, but it still has its limitations. The main problems are long running time and information lost in preprocessing. The problems in the detection results are mainly manifested in the occurrence of breaks in the detected bankline.

The main reason for the long running time of the proposed algorithm is that the proposed algorithm is a combination of multiple methods and has many steps, so the running speed is slow. The proposed algorithm loses part of the information in the preprocessing process. The main reason is that the non-local mean preprocessing suppresses the speckle of GF-3 SAR image while also filtering out some image information. As a result, several bankline information is lost and cannot be extracted during the following stages. In Fig. 4, the bankline detected by the proposed algorithm has a fracture problem. The main reason is that the proposed algorithm uses the OTSU algorithm to adaptively adjust the gray level of the image during the binarization and bankline thinning stages. At this time, the threshold calculated can ensure that the gray value variance between the bankline area and the background area is the largest, but it cannot guarantee that the threshold can separate the river bank completely and accurately. Therefore, it will cause the detected bankline to break, as shown in Figs. 4 and 5.

In short, the proposed algorithm is very suitable for detecting bankline information under strong speckle interference in GF-3 SAR images, but it needs to be improved, such as from aspects of image preprocessing.

## Conclusions

In view of the shortcomings of low detection accuracy and sensitivity to speckle of the traditional difference operators, we propose a bankline detection algorithm for GF-3 SAR images based on shearlet. The proposed algorithm makes full use of the characteristics of the optimal sparsity and strong directional position of shearlet, and the detection performance is well. Firstly, the non-local mean filter is used to preprocess GF-3 SAR images, so as to reduce the interference of speckle on bankline detection. Secondly, shearlet is used to detect the bankline. Finally, morphological processing is used to refine the bankline and further eliminate the false bankline, leading to ideal bankline detection results. We use three different regions of GF-3 SAR images to verify the effectiveness of the proposed algorithm, and compare it with other five algorithms to analyze the performance of the proposed algorithm in four aspects: anti-speckle, smoothness, completeness and running speed. Experimental results show that the proposed algorithm is suitable for bankline detection in GF-3 SAR images, and can ensure that the detected bankline is continuous, and smooth, and the position is consistent with the original image. Therefore, it is a very suitable algorithm for bankline detection in GF-3 SAR images.

## Supplemental Information

10.7717/peerj-cs.611/supp-1 Supplemental Information 1 The code of bankline detection program. Based on the excellent multi-scale, directionality and the optimal sparsity of the shearlet, a bankline detection program based on shearlet is proposed. Firstly, we use non-local means filter to preprocess GF-3 SAR image, so as to reduce the interference of speckle on bankline detection. Secondly, shearlet is used to detect the bankline of the image. Finally, morphological processing is used to refine the bankline and further eliminate the false bankline caused by the speckle, so as to obtain the ideal bankline detection results. Experimental results show that the proposed method can effectively overcome the interference of speckle, and can detect the bankline information of GF-3 SAR image completely and smoothly.

## Additional Information and Declarations

### Data Availability

The following information was supplied regarding data availability: Data are available at China Centre for Resources Satellite Data and Application, and the image data used in the experiment in the database is available at GitHub: https://github.com/Donlting/GF-3-SAR-images. The code of bankline detection program is in the Supplemental File.
